# Editorial: One-dimensional nanostructures and their inspired applications in catalysis

**DOI:** 10.3389/fchem.2022.1113117

**Published:** 2022-12-23

**Authors:** Sheng Cao

**Affiliations:** MOE Key Laboratory of New Processing Technology for Non-ferrous Metals and Materials, School of Physical Science and Technology, Guangxi University, Nanning, China

**Keywords:** photocatalysis, electrocatalysis, 1D nanostructures, rational design, synthesis

One-dimensional (1D) nanomaterials exist in the form of wire, fiber, ribbon, tube, and rod, *etc*. Due to their unique physical and chemical properties and wide application potential, they have aroused widespread interest. Especially in the application of photocatalysis and electrocatalysis, 1D nanomaterials show good stability due to their special geometric structure, which provides a good platform for catalytic reactions. Meanwhile, 1D nanomaterials have the merits of large specific surface area, high electron-hole separation efficiency, strong light absorption ability, and rich exposed active centers, which accelerate the charge transfer and charge separation in the catalytic reaction, thereby improving the efficiency of the catalytic reaction. For these reasons, researchers have been actively designing and developing new 1D nanomaterials and exploring the basic characteristics of new catalytic materials with well-designed structures. Therefore, this Research Topic aims to highlight the latest progress in the design and synthesis of 1D nanostructures and their inspiring applications in catalysis.

In this Research Topic, representative types of 1D nanomaterial design strategies are discussed in detail to provide reasonable solutions for improving catalytic performance. They include Pd-Co_3_O_4_ nanofibers prepared based on electrospinning and subsequent heat treatment (Wang et al.) and new Bi_2_O_3_ nanostructures prepared by microwave-assisted synthesis (Yahyazadehfar et al.). In addition to the original research articles published here, our Research Topic also includes a review article. Yuan et al. reviewed the application progress of 1D nanomaterials in photocatalysis and electrocatalysis, especially the single molecule and single particle research of 1D nanomaterials. These studies prove that the single molecule and single particle research of 1D nanomaterials can improve the understanding of complex processes in the field of catalysis, and provide insights for optimizing the performance of catalytic systems. This review systematically introduces the Frontier fields that open the way for 1D nanomaterial catalysis.

In the electrocatalytic application of 1D nanomaterials, Wang et al. synthesized palladium doped Co_3_O_4_ on carbon nanofiber materials by electrospinning and subsequent heat treatment and used it as a self-supporting electrode (Pd-Co_3_O_4_@CNF). Benefiting from the rich active sites and high specific surface area of the 1D nanostructure, Pd-Co_3_O_4_@CNF works as a bifunctional oxygen electrode and shows extraordinary oxygen reduction reaction and oxygen evolution reaction performance in alkaline solution. Compared with commercial catalysts based on zinc-air batteries, Pd-Co_3_O_4_@CNF electrode exhibit superior charge-discharge capacity and stability. This study provides an efficient and scalable convenient method to synthesize high-performance self-supporting 1D nanostructured electrodes and proposes a general strategy for manufacturing flexible self-supporting electrodes which can be applied to energy storage and conversion devices.

Moving to the field of catalytic synthesis, Yahyazadehfar et al. proposed effective, cost-effective, and rapid microwave-assisted synthesis methods to synthesize the novel nanostructures of Bi_2_O_3_ under environmental conditions. The average particle size distribution of the final products is 85 nm, and the surface area is 783 m^2^/g. The study confirmed that this Bi_2_O_3_ nanostructure has excellent thermodynamic stability. The prepared Bi_2_O_3_ nanostructure is used as a catalyst for the synthesis of arylmethylidene barbituric acid derivatives. After the catalyst is added to the water medium, all reactions are completed within 2–3 min at room temperature. The main advantages of this method are strong practicability, easy availability of raw materials, low cost, and reusable catalyst. In addition, the catalyst synthesis process can be carried out in a water medium for a short time with a medium to high yield. The results open a new window for the development of new catalysts with practical application value.

Although the papers collected here provide significant insights, the preparation and catalytic mechanism of 1D nanomaterials still face significant challenges due to the subtle effects of size, shape, and structural defects. In the future, more attention should be paid to the design and preparation of new one-dimensional nanostructures, and simple preparation methods for large-scale production should be developed. At the same time, the growth kinetics of 1D nanomaterials should be further understood through *in situ* characterization technology. It is also necessary to strengthen the understanding of the electron transport and separation process in the catalytic process of 1D nanomaterials by means of ultrafast spectral analysis and testing technology, and the relationship between the size, shape, structural defects of 1D nanomaterials and their catalytic performance need to be established in detail. To improve the testing efficiency of catalytic performance of 1D nanostructured materials, high-speed acquisition and automated data analysis are required. Mathematical tools such as big data processing and machine learning maybe will shine brightly in these fields.

We thank all the authors for their meaningful work, as well as all the reviewers for their profound suggestions and constructive comments. It is hoped that this Research Topic will stimulate the research on the discovery and design of new 1D nanomaterials in the future and promote the in-depth development of efficient catalytic applications. We expect that these efforts will pave the way for green growth and sustainable development.

